# Comparison of vacuum static quadrupolar metrics

**DOI:** 10.1098/rsos.170826

**Published:** 2018-05-09

**Authors:** Francisco Frutos-Alfaro, Hernando Quevedo, Pedro A. Sanchez

**Affiliations:** 1 School of Physics and Space Research Center, University of Costa Rica, San José, Costa Rica; 2 Instituto de Ciencias Nucleares, Universidad Nacional Autónoma de México, AP 70543, Ciudad de México 04510, Mexico; 3 Dipartimento di Fisica and ICRA, Università di Roma ‘La Sapienza’, 00185 Roma, Italy; 4 Institute of Experimental and Theoretical Physics, Al-Farabi Kazakh National University, Almaty, Kazakhstan

**Keywords:** quadrupole moment, naked singularities, vacuum metrics

## Abstract

We investigate the properties of static and axisymmetric vacuum solutions of Einstein equations which generalize the Schwarzschild spherically symmetric solution to include a quadrupole parameter. We test all the solutions with respect to elementary and asymptotic flatness and curvature regularity. Analysing their multipole structure, according to the relativistic invariant Geroch definition, we show that all of them are equivalent up to the level of the quadrupole. We conclude that the *q*-metric, a variant of the Zipoy–Voorhees metric, is the simplest generalization of the Schwarzschild metric, containing a quadrupole parameter.

## 1. Introduction

Most applications of Einstein’s gravity theory follow from the investigation of exact solutions of the corresponding field equations. In the case of relativistic astrophysics, asymptotically flat solutions in empty space are of particular importance in order to describe the physical properties of the exterior field of compact objects [1]. From a physical point of view, it is sufficient in this case to limit ourselves to static and stationary solutions which are axially symmetric. In addition, it is appropriate to classify them in accordance with certain criteria which permits a comparison of their main properties. Using the analogy with Newtonian gravity, we propose to classify them in terms of their multipole moments.

The problem of defining invariant multipole moments in general relativity was first solved by Geroch and Hansen (GH) [2,3], who proposed definitions for mass and spin multipoles of asymptotically flat space–times in vacuum. Moreover, Simon and Beig [4] and Thorne [5] defined relativistic multipole moments for stationary space–times. A proof of the equivalence between the GH moments and the Thorne moments for stationary systems was provided by Gürsel [6]. An elegant method to derive explicit expressions for the multipole moments of a given stationary and axially symmetric space–time with asymptotic flatness was found by Fodor *et al.* [7] using the Ernst formalism. This FHP method was generalized by Hoenselaers & Perjés [8]. Finally, Ryan [9] found an alternative method for deriving the relativistic multipole moments which has been intensively applied in relativistic astrophysics.

Although for the study of the gravitational field of relativistic compact objects it is necessary to consider stationary solutions that take into account the rotation of the source, in this work we will focus on the study of the static case to explore in detail the physical properties of the solutions which then will be generalized to the case of stationary fields. From a physical point of view, the most important multipoles of a mass distribution are the monopole and the quadrupole; in this work, we will focus our analysis on mainly these two multipoles.

The first solution with only monopole moment was derived by Schwarzschild in 1916 [10], just a couple of months after the publication of the theory of general relativity. In 1917, Weyl found a class of static and axisymmetric solutions to the vacuum Einstein field equations [11,12]. The first static solution with quadrupole moment which includes the Schwarzschild metric as special case was found by Erez & Rosen in 1959 [13,14]. This quadrupolar solution was generalized to include an infinite mumber of multipole moments by Quevedo [15]. In 1966 and 1970, Zipoy [16] and Voorhees [17] found a transformation which allows us to generate new static solutions from known solutions. In particular, applying this transformation to the Schwarzschild metric, one obtains a new solution which, after a redefinition of the Zipoy–Voorhees parameter, was interpreted as the simplest static solution which generalizes the Schwarzschild metric and includes a quadrupole moment (*q*-metric) [18]. In 1985, Gutsunaev and Manko [19] found an exact solution with monopole and quadrupole moments which was shown in Quevedo [20] to have the same quadrupole as in the Erez–Rosen metric, but different contributions to higher relativistic multipole moments. In 1990, Manko [21] found a quadrupolar metric which can be interpreted as the nonlinear combination of the Schwarzschild monopole solution with the quadrupolar term of the Weyl solution. In 1994, Hernández-Pastora & Martín [22] derived two exact solutions with different monopole–quadrupole structures.

To our knowledge, the above list includes all known static and asymptotically flat solutions of Einstein’s equations in empty space. The main goal of the present work is to investigate the most important physical properties of these solutions. In particular, we will analyse the elementary flatness condition, curvature singularities, multipole moments structure and the relationships between them.

This paper is organized as follows. In §2, we present the general line element for static axisymmetric space–times and the corresponding vacuum field equations, and review the most general aymptotically flat solution in cylindrical coordinates discovered by Weyl. In §3, we present the solutions that contain the Schwarzschild space–time as a particular case and an additional parameter which determines the quadrupole of the gravitational source. Then, in §4, we investigate the conditions that the solutions must satisfy in order to be able to describe the exterior gravitational field of compact objects. Section 5 is devoted to the study of the multipole structure of the solutions. Finally, in §6, we discuss our results and present some initiatives for future works.

## 2. General properties of static and axisymmetric vacuum solutions

Although there exist in the literature many suitable coordinate systems, static axisymmetric gravitational fields are usually described in cylindrical coordinates (*t*,*ρ*,*z*,*φ*), following the seminal work of Weyl. Stationarity implies that there exists a time-like Killing vector field with components $\delta_{t}^{\alpha }$, i.e. *t* can be chosen as the time coordinate and the metric does not depend on time, ∂*g*_*αβ*_/∂*t*=0. Axial symmetry, in addition, implies the existence of a space-like Killing vector field with components $\delta_{\varphi }^{\alpha }$, which commutes with the time-like Killing vector. The coordinates can then be chosen such that ∂*g*_*αβ*_/∂*φ*=0, and the axis of symmetry corresponds to *ρ*=0. Furthermore, if we assume that the time-like Killing vector is hypersurface-orthogonal, the space–time is static, i.e. it is invariant with respect to the transformation φ→−φ.

Furthermore, using the properties of staticity and axial symmetry, together with the vacuum field equations, for a general metric of the form *g*_*αβ*_=*g*_*αβ*_(*ρ*,*z*), it is possible to show that the most general line element for this type of gravitational field can be written in the Weyl–Lewis–Papapetrou form [1,11,23,24] as

2.1 $$ds^{2}=e^{2\psi }\;dt^{2}-e^{-2\psi }[e^{2\gamma }(d\rho^{2}+dz^{2})+\rho^{2}\;d\varphi^{2}],$$

where *ψ* and *γ* are functions of *ρ* and *z* only. The vacuum field equations can be reduced to the following set of independent differential equations:

2.2 $$\psi_{\rho \rho }+\frac{1}{\rho }\psi_{\rho }+\psi_{zz}=0$$

and

2.3 $$\gamma_{\rho }=\rho (\psi_{\rho }^{2}-\psi_{z}^{2}),\;\gamma_{z}=2\rho \psi_{\rho }\psi_{z},$$

where *ψ*_*ρ*_=∂*ψ*/∂*ρ*, etc. We see that the main field equation (2.2) corresponds to the linear Laplace equation for the metric function *ψ*. Furthermore, the solution for the function *γ* can be obtained by quadratures once the function *ψ* is known.

The general solution of Laplace’s equation is known and, if we demand additionally asymptotic flatness, we obtain the Weyl solution [1,11]

2.4 $$\psi =\sum_{n=0}^{\infty }\frac{a_{n}}{{\left(\rho^{2}+z^{2}\right)}^{(n+1)/2}}P_{n}(\cos\theta ),\;\cos\theta =\frac{z}{\sqrt{\rho^{2}+z^{2}}},$$

where *a*_*n*_ (*n*=0,1,…) are arbitrary real constants and $P_{n}(\cos\theta )$ represents the Legendre polynomials of degree *n*. The expression for the metric function *γ* can be obtained from the two first-order differential equations (2.3). Then

2.5 $$\gamma =-\sum_{n,m=0}^{\infty }\frac{a_{n}a_{m}(n+1)(m+1)}{(n+m+2){\left(\rho^{2}+z^{2}\right)}^{(n+m+2)/2}}(P_{n}P_{m}-P_{n+1}P_{m+1}).$$

As this is the most general static, axisymmetric, asymptotically flat vacuum solution, it must contain all known solutions of this class. In particular, one of the most interesting special solutions, which is Schwarzschild’s spherically symmetric black hole space–time, must be included as a special case. To see this, we must choose the constants *a*_*n*_ in such a way that the infinite sum (2.4) converges to the Schwarzschild solution in cylindrical coordinates. A straightforward computation shows that

2.6 $$a_{2n}=-\frac{m^{2n+1}}{2n+1},\;a_{2n+1}=0,$$

where *m* is the mass parameter [25]. Clearly, this representation is not appropriate to handle the Schwarzschild metric.

It turns out that to investigate the properties of solutions with multipole moments, it is convenient to use prolate spheroidal coordinates (*t*,*x*,*y*,*φ*) in which the line element can be written as

2.7 $$ds^{2}=e^{2\psi }\;dt^{2}-\sigma^{2}e^{-2\psi }\;\left[e^{2\gamma }(x^{2}-y^{2})\left(\frac{dx^{2}}{x^{2}-1}+\frac{dy^{2}}{1-y^{2}}\right)+(x^{2}-1)(1-y^{2})\;d\varphi^{2}\right],$$

where

2.8 $$x=\frac{r_{+}+r_{-}}{2\sigma },\;(x^{2}\geq 1),\;y=\frac{r_{+}-r_{-}}{2\sigma },\;(y^{2}\leq 1)$$

and

2.9 $$r_{\pm }^{2}=\rho^{2}+{\left(z\pm \sigma \right)}^{2},\;\sigma =\mathrm{const}.,$$

and the metric functions *ψ*, and *γ* depend on *x* and *y*, only. In this coordinate system, the field equations become

2.10 $${\left[(x^{2}-1)\psi_{x}\right]}_{x}+{\left[(1-y^{2})\psi_{y}\right]}_{y}=0$$

and

2.11 $$\begin{array}{rl} \gamma_{x} & =\left(\frac{1-y^{2}}{x^{2}-y^{2}}\right)\;[x(x^{2}-1)\psi_{x}^{2}-x(1-y^{2})\psi_{y}^{2}-2y(x^{2}-1)\psi_{x}\psi_{y}],\\ \;\text{and}\;\gamma_{y} & =\left(\frac{x^{2}-1}{x^{2}-y^{2}}\right)\;[y(x^{2}-1)\psi_{x}^{2}-y(1-y^{2})\psi_{y}^{2}+2x(1-y^{2})\psi_{x}\psi_{y}]. \end{array}\}$$

The simplest physically meaningful solution to the above system of differential equations is the Schwarzschild solution

2.12 $$\psi_{S}=\frac{1}{2}\ln\;\left(\frac{x-1}{x+1}\right)\;\mathrm{and}\;\gamma_{S}=\frac{1}{2}\ln\;\left(\frac{x^{2}-1}{x^{2}-y^{2}}\right),$$

which takes the standard form in spherical coordinates with *x*=*r*/*m*−1, y=cos⁡θ and *σ*=*m*. In principle, there could be an infinite number of exact solutions to the above equations. Not all of them, however, can be physically meaningful, in particular if we demand that they should describe the exterior field of realistic compact objects. To this end, it is necessary that the solutions satisfy the conditions of asymptotic flatness, elementary flatness and regularity.

Asymptotic flatness means that, at spatial infinity, the solution reduces to the Minkowski metric, indicating that the gravitational field far away from the source is practically negligible. This is a consequence of the long-range property of the gravitational interaction. In the case of the static metric in prolate spheroidal coordinates (2.7), this condition implies that

2.13 $$\lim_{x\to \infty }\psi =\mathrm{const}.\;\mathrm{and}\;\lim_{x\to \infty }\gamma =\mathrm{const}.,$$

where the constants can be set equal to zero by a suitable rescaling of the coordinates.

Elementary flatness is necessary in order to guarantee that near the rotation axis the geometry is Lorentzian, i.e. there are no conical singularities on the axis [1]. This condition can be expressed in an invariant manner by using the space-like Killing vector field $\eta^{\alpha }=\delta_{\varphi }^{\alpha }$ as

2.14 $$\lim_{\rho \to 0}\frac{{\left(\eta^{\alpha }\eta_{\alpha }\right)}^{,\beta }{\left(\eta^{\alpha }\eta_{\alpha }\right)}_{,\beta }}{4(\eta^{\alpha }\eta_{\alpha })}=1.$$

A direct computation by using the general line element in prolate spheroidal coordinates shows that the elementary flatness condition is equivalent to demanding that

2.15 $$\lim_{y\to \pm 1}\gamma =0,$$

independently of the value of the spatial coordinate *x*.

Finally, the regularity condition implies that the solution must be free of curvature singularities outside a region located near the origin of coordinates so that it can be covered by an interior solution. Curvature singularities can be detected by analysing the behaviour of curvature invariants. In general, the Riemann curvature tensor in four dimensions possesses 14 independent invariants. In the case of vacuum space–times, however, the Riemann tensor coincides with the Weyl tensor that has only four invariants which can be expressed as [26]

2.16 $$\begin{array}{rl} K & =I_{1}=R_{\alpha \beta \gamma \delta }R^{\alpha \beta \gamma \delta },\;I_{2}=*R_{\alpha \beta \gamma \delta }R^{\alpha \beta \gamma \delta },\\ \;\text{and}\;I_{3} & =R_{\alpha \beta \gamma \delta }R^{\gamma \delta \lambda \tau }R_{\lambda \tau }^{\alpha \beta },\;I_{4}=*R_{\alpha \beta \gamma \delta }R^{\gamma \delta \lambda \tau }R_{\lambda \tau }^{\alpha \beta }, \end{array}\}$$

where the dual is defined as

2.17 $$*R_{\alpha \beta \gamma \delta }=\frac{1}{2}\epsilon_{\alpha \beta \lambda \tau }R_{\gamma \delta }^{\lambda \tau },$$

with *ϵ*_*αβλτ*_ being the Levi-Civita symbol. The quadratic invariants *K*=*I*_1_ and *I*_2_ are usually known as the Kretschmann and the Chern–Pontryagin scalars, respectively. If any one of the four invariants happens to diverge at some particular place, it is said that there exists a curvature singularity at that place.

In the next section, we will investigate the properties of several exact solutions with monopole and quadrupole moment. In particular, we will find out if they satisfy all the conditions to be physically relevant in the sense that they can be used to describe the exterior gravitational field of compact objects.

## 3. Static vacuum metrics with quadrupole

As mentioned in the last section, the Weyl metric can be considered as the most general static and axisymmetric solution which contains an infinite number of parameters, representing all the multipole moments. Therefore, a particular choice of parameters could represent a solution with only mass and quadrupole. However, such a form of a metric with an infinite number of parameters is not very suitable to be applied in the case of realistic sources like compact astrophysical objects. For this reason, we consider now metrics which include only two independent parameters that can be interpreted as mass and quadrupole.

In 1959, Erez & Rosen [14] presented a solution which generalizes the Schwarzschild metric and contains an additional parameter *q*. In this case, the function *ψ* can be expressed as

3.1 $$\psi_{\mathrm{ER}}=\frac{1}{2}\ln\;\left(\frac{x-1}{x+1}\right)+\frac{1}{2}q(3y^{2}-1)\left[\frac{1}{4}(3x^{2}-1)\ln\;\left(\frac{x-1}{x+1}\right)+\frac{3}{2}x\right].$$

The corresponding function *γ*_ER_ cannot be expressed in a compact form and is given explicitly in appendix A. This solution was obtained by using the method of separation of variables for the function *ψ*. An explicit generalization which contains higher multipole moments was presented in 1989 by Quevedo [15] by using the same method.

In 1985, Gutsunayev & Manko [19] found a new static solution for the function *ψ* which is given by

3.2 $$\psi_{\mathrm{GM}}=\frac{1}{2}\ln\;\left(\frac{x-1}{x+1}\right)+q\frac{x}{{\left(x^{2}-y^{2}\right)}^{3}}(x^{2}-3x^{2}y^{2}+3y^{2}-y^{4})$$

and the function *γ*_GM_ is given explicitly in appendix A. This solution was found by applying a particular differential operator to the Schwarzschild metric. This method was shown to be based upon the property that in Cartesian coordinates the derivatives of a harmonic function are also harmonic functions [20].

In 1990, Manko [21] found a different static solution in the form

3.3 $$\psi_{M}=\frac{1}{2}\ln\;\left(\frac{x-1}{x+1}\right)+q\frac{3x^{2}y^{2}-x^{2}-y^{2}+1}{2{\left(x^{2}+y^{2}-1\right)}^{5/2}},$$

which leads to a particular function *γ*_M_ given in appendix A. The first term of this solution corresponds to the Schwarzschild metric, whereas the second term coincides with the quadrupolar term of the general Weyl solution in prolate spheroidal coordinates.

Furthermore, in 1994, Hernández-Pastora & Martín [22] derived two different exact solutions which can be written as

3.4 $$\psi_{\mathrm{HM}1}=\frac{1}{2}\ln\;\left(\frac{x-1}{x+1}\right)-\frac{5}{8}q\left[\frac{1}{4}((3x^{2}-1)(3y^{2}-1)-4)\ln\;\left(\frac{x-1}{x+1}\right)+\frac{2x}{\left(x^{2}-y^{2}\right)}-\frac{3}{2}x(3y^{2}-1)\right]$$

and

3.5 $$\begin{array}{rl} \psi_{\mathrm{HM}2} & =\psi_{\mathrm{HM}1}-\frac{5}{32}q^{2}\left[\left(33+90P_{2}(x)P_{2}(y)-\frac{153}{2}P_{4}(x)P_{4}(y)\right)\ln\;\left(\frac{x-1}{x+1}\right)-135xP_{2}(y\right)\\ & \;-\frac{153}{24}x(55-105x^{2})P_{4}(y)-\frac{x}{x^{2}-y^{2}}\left(33-\frac{5}{{\left(x^{2}-y^{2}\right)}^{2}}(3x^{2}y^{2}+y^{4}-x^{2}-3y^{2})\right)], \end{array}$$

where

3.6 $$P_{2}(y)=\frac{1}{2}(3y^{2}-1)$$

and

3.7 $$P_{4}(y)=\frac{1}{8}(35y^{4}-30y^{2}+3).$$

Here we have corrected several typos which are present in the original publications. The corresponding functions *γ*_*HM*1_ and *γ*_*HM*2_ have quite a complicated structure which we present explicitly in appendix A.

Finally, in 1966 and 1970, Zipoy [16] and Voorhees [17], respectively, found a particular symmetry of the vacuum field equations, and derived a transformation which can be used to generate new solutions from known solutions. In the case of the Schwarzschild metric, the new solution can be expressed simply as

3.8 $$\psi_{\mathrm{ZV}}=\frac{1}{2}\delta \ln\;\left(\frac{x-1}{x+1}\right)\;\mathrm{and}\;\gamma_{\mathrm{ZV}}=\frac{1}{2}\delta^{2}\ln\;\left(\frac{x^{2}-1}{x^{2}-y^{2}}\right),$$

where *δ* is an arbitrary real constant. This solution is also known as the *δ*-metric of the *γ*-metric for notational reasons [27]. Later on, in 2011, this metric was reinterpreted as a quadrupolar metric and renamed as the *q*-metric [18] which in spherical coordinates can be transformed into the simple form

3.9 $$\begin{array}{rl} ds^{2} & ={\left(1-\frac{2m}{r}\right)}^{1+q}\;dt^{2}-{\left(1-\frac{2\;m}{r}\right)}^{-q}\\ & \;\times \left[{\left(1+\frac{m^{2}\sin^{2}\theta }{r^{2}-2mr}\right)}^{-q(2+q)}\left(\frac{dr^{2}}{1-2m/r}+r^{2}d\theta^{2}\right)+r^{2}\sin^{2}\theta \;d\varphi^{2}\right]. \end{array}$$

It is easy to see that all the above solutions represent a generalization of the Schwarzschild metric which is obtained in the limiting case *q*→0. To our knowledge, the solutions presented above are the only exact solutions that generalize the Schwarzschild monopole solution and satisfy the conditions expected from a metric that describes a realistic gravitational field.

## 4. Physical conditions

All the solutions presented in the last section are asymptotically flat because at spatial infinity they behave as

4.1 $$\psi_{0}=\lim_{x\to \infty }\psi =0\;\mathrm{and}\;\gamma_{{\;}_{0}}=\lim_{x\to \infty }\gamma =0,$$

which determine the Minkowski metric, independently of the value of *y*. Note, moreover, that this condition is satisfied for all finite values of the independent parameters *m* and *q*. This means that, for any finite values of the monopole and quadrupole moments, the solutions presented in the last section are asymptotically Minkowski.

As mentioned above, the condition that no conical singularities exist on the symmetry axis (2.14) in prolate spheroidal coordinates becomes

4.2 $$\lim_{y\to \pm 1}\gamma =0.$$

An inspection of the *γ* function for the Erez–Rosen, Gutsunayev–Manko and Manko solutions and the *q*-metric, mentioned in the last section, shows that this condition is always satisfied, independently of the value of *x*, indicating that all of them are elementary flat. In the case of the Hernández–Martín (HM) solutions, however, a direct computation shows that they are elementary flat only for positive values of the coordinate *x*. In spherical coordinates, this means that the HM solutions are well defined only outside the radius *r*=2*m*. A geometric and physical analysis inside the horizon *r*=2*m* is possible only by considering the presence of conical singularities along the symmetry axis.

We now analyse the regularity condition by using first the Kretschmann scalar *K*=*R*_*αβγδ*_*R*^*αβγδ*^. Fist, we consider the Schwarzschild metric (2.12) for which we obtain

4.3 $$K_{S}=\frac{48}{m^{4}{\left(x+1\right)}^{6}}.$$

This expression is singular only for *x*=−1 (*r*=0), indicating the well-known fact that the Schwarzschild space–time is singular only at the origin of coordinates.

Another example of a solution that can be investigated analytically is the *q*-metric. In this case, all the calculations can be performed explicitly and the resulting Kretschmann scalar reads

4.4 $$K_{q}=\frac{48}{\sigma^{4}}{\left(q+1\right)}^{2}\frac{p(x,y;q)}{{\left(x+1\right)}^{2(q^{2}+3q+3)}{\left(x-1\right)}^{2(q^{2}+q+1)}{\left(x^{2}-y^{2}\right)}^{-2q^{2}-4q+1}},$$

where

4.5 $$\begin{array}{rl} p(x,y;q) & ={\left(x-1\right)}^{2}(x^{2}-y^{2})-2q{\left(x-1\right)}^{2}(x+y^{2})+q^{2}\left[(2-y^{2})x^{2}-3(1-y^{2})x+\frac{1}{2}(4-7y^{2})\right]\\ & \;-q^{3}\left(x-\frac{4}{3}\right)(1-y^{2})+\frac{1}{3}q^{4}(1-y^{2}). \end{array}$$

First, we see that, for all values of *q*, there is always a singularity at *x*=−1. Moreover, we have two possible divergences at *x*=1 and *x*=±*y*. These divergent factors can only be cancelled by the function *p*, but it does not vanish for *x*=1 or *x*=±*y* for arbitrary values of *q*, except for *q*=−2. In this case, one has *p*(*x*,*y*;−2)=(*x*+1)^2^(*x*^2^−*y*^2^) so that

4.6 $$K_{q=-2}=\frac{48}{\sigma^{4}{\left(x-1\right)}^{6}},$$

which diverges for *x*=1. For other values of the parameter *q*, the Kretschmann scalar of the *q*-metric diverges at *x*=±1 and *x*=±*y*, as far as the exponents of the corresponding factors are negative. The exponents of the factors *x*+1 and *x*−1 are negative definite, but the exponent of the factor *x*^2^−*y*^2^ vanishes for $q=-1+\sqrt{\frac{3}{2}}$ and $q=-1-\sqrt{\frac{3}{2}}$.

Consequently, the Kretschmann scalar of the *q*-metric diverges at *x*=−1 for *q*≠−2, at *x*=1 for *q*≠0 and at *x*=±*y* for $q\in (-1-\sqrt{\frac{3}{2}},-1+\sqrt{\frac{3}{2}})$ restricted to *q*≠0 and *q*≠−2. An additional restriction to the value of the parameter *q* is imposed by assuming *σ*>0 and requiring its mass monopole to be positive. We will see in the next section that this physical condition implies that *q*>−1, leading to the conclusion that the singularity at *x*=−1 is always present.

The investigation of the remaining quadrupolar solutions is much more complicated. In appendix B, we present as an example the explicit analysis of the Erez–Rosen metric. The results of our analysis are summarized in table 1, where we include the Schwarzschild solution for comparison, and use spherical coordinates with *x*=*r*/*m*−1 and y=cos⁡θ. The bold-faced radii represent singularities that are present, independently of the value of the parameters *m*, *q* and the coordinate *θ*. The remaining radii represent singularities which are not always present, but depend on the value of *q* or the coordinate *θ*. We see that only the *q*-metric is characterized by a completely singular horizon at *r*=2*m*, representing the outermost singularity, which is the only one that can be observed by an exterior observer. In the remaining cases, the Schwarzschild horizon remains partially regular, implying that for certain values of *q*, it is possible to observe the singularity located at the origin of coordinates.

**Table 1.** Singularities of space–times with monopole and quadrupole moments. Bold-faced values are naked singularities which exist for all values of the parameters *m*, *q* and *θ*. Other singularities exist only for particular values of these parameters.

static metric	naked singularites
Schwarzschild	***r*=0**
*q*-metric	r=0, m(1±cosθ), 2m
Erez–Rosen	r=0, m(1±cosθ), 2m
Gutsunayev–Manko	r=0, m(1±cosθ), 2m
Manko	r=0, m(1±cosθ), 2m
Hernández–Martín 1 and 2	r=0, m(1±cosθ), 2m

Finally, we mention that the analysis of the remaining three curvature invariants does not lead to additional singularities.

## 5. Multipole moments

Using the original definition formulated by Geroch [2], the calculation of multipole moments is quite laborious. Fodor *et al.* [7] found a relation between the Ernst potential [28,29] and the multipole moments which facilitates the computation. In the case of static axisymmetric space–times, the Ernst potential is defined as

5.1 $$\xi (x,y)=\frac{1-e^{2\psi }}{1+e^{2\psi }}.$$

The idea is that the multipole moments can be obtained explicitly from the values of the Ernst potential on the axis by using the following procedure. On the axis of symmetry *y*=1, we can introduce the inverse of the Weyl coordinate *z* as

5.2 $$\tilde{z}=\frac{1}{z}=\frac{1}{mx},\;\mathrm{with}\;\sigma =m.$$

If we now introduce the inverse potential as

5.3 $$\tilde{\xi }(\tilde{z},1)=\frac{1}{\tilde{z}}\xi (\tilde{z},1),$$

the multipole moments can be calculated as

5.4 $$M_{n}=m_{n}+d_{n},\;m_{n}=\frac{1}{n!}{\frac{d^{n}\tilde{\xi }(\tilde{z},1)}{d{\tilde{z}}^{n}}|}_{\tilde{z}=0},$$

where the additional terms *d*_*n*_ must be determined from the original Geroch definition. The main point now is that the first term *m*_*n*_ is completely determined by the *n*-th derivative of the inverse Ernst potential $\tilde{\xi }$, whereas the second term *d*_*n*_ depends on the derivatives of order less than *n*, so that the moment $M_{n}$ can be calculated explicitly once all the derivatives of order *n* or less are known. In appendix C, we include the explicit expressions for the first 10 additional terms.

In this manner, it is easy to show that for the Schwarzschild space–time the multipole moments are given as

5.5 $$M_{0}=m,\;M_{k}=0,\;(k\geq 1),$$

a result which is in accordance with the physical interpretation of the Schwarzschild metric obtained by using other methods.

For the Erez–Rosen metric, we obtain

$$\begin{array}{rl} M_{0} & =m,\\ M_{2} & =Q,\\ M_{4} & =-\frac{2}{7}Qm^{2},\\ M_{6} & =-\frac{8}{231}Qm^{4}(1+3q),\\ M_{8} & =-\frac{8}{3003}Qm^{6}\left(2-\frac{74}{15}q+\frac{84}{45}q^{2}\right)\\ \;\mathrm{and}\;M_{10} & =\frac{32}{3927}Qm^{8}\left(-\frac{28}{247}+\frac{37}{57}q+\frac{1124}{741}q^{2}\right), \end{array}$$

where *Q*=2*qm*^3^/15.

For the Gutsunayev–Manko metric, we obtain

$$\begin{array}{rl} M_{0} & =m,\\ M_{2} & =Q,\\ M_{4} & =\frac{6}{7}Qm^{2},\\ M_{6} & =\frac{8}{231}Qm^{4}(14-45q),\\ M_{8} & =\frac{8}{3003}Qm^{6}(84-1282q-420q^{2})\\ \;\mathrm{and}\;M_{10} & =\frac{32}{3927}Qm^{8}\left(\frac{2772}{247}-\frac{1343804}{2717}q-\frac{2550}{19}q^{2}\right), \end{array}$$

where *Q*=2*qm*^3^.

For the Manko solution, we obtain

$$\begin{array}{rl} M_{0} & =m,\\ M_{2} & =Q=-m^{3}q,\\ M_{4} & =-\frac{8}{7}Qm^{2},\\ M_{6} & =\frac{1}{231}Qm^{4}(180q+133),\\ M_{8} & =-\frac{2}{3003}Qm^{6}(420q^{2}+2182q+357)\\ \;\mathrm{and}\;M_{10} & =\frac{1}{969969}Qm^{8}(1379100q^{2}+1277710q+85701). \end{array}$$

For the first Hernández–Martín metric, we obtain

$$\begin{array}{rl} M_{0} & =m,\\ M_{2} & =Q,\\ M_{4} & =0,\\ M_{6} & =-\frac{60}{77}qQm^{4},\\ M_{8} & =-\frac{4}{3003}qQm^{6}(265+210q),\\ \;\mathrm{and}\;M_{10} & =\frac{4}{3927}qQm^{8}\left(-\frac{34790}{247}+\frac{769125}{1729}q\right) \end{array}$$

and for the second Hernández–Martín solution

$$\begin{array}{rl} M_{0} & =m,\\ M_{2} & =Q,\\ M_{4} & =0,\\ M_{6} & =0,\\ M_{8} & =-\frac{40}{143}q^{2}Qm^{6}\\ \;\mathrm{and}\;M_{10} & =-\frac{42140}{46189}q^{2}Qm^{8}, \end{array}$$

where *Q*=*qm*^3^.

Finally, for the *q*-metric we get

$$\begin{array}{rl} M_{0} & =\delta m,\\ M_{2} & =\frac{1}{3}\delta m^{3}(1-\delta^{2}),\\ M_{4} & =\delta m^{5}\left(\frac{19}{105}\delta^{4}-\frac{8}{21}\delta^{2}+\frac{1}{5}\right),\\ M_{6} & =\delta m^{7}\left(-\frac{389}{3465}\delta^{6}+\frac{23}{63}\delta^{4}-\frac{457}{1155}\delta^{2}+\frac{1}{7}\right),\\ M_{8} & =\delta m^{9}\left(\frac{257}{3465}\delta^{8}-\frac{44312}{135135}\delta^{6}+\frac{73522}{135135}\delta^{4}-\frac{54248}{135135}\delta^{2}+\frac{1}{9}\right)\\ \;\mathrm{and}\;M_{10} & =\delta m^{11}\left(-\frac{443699}{8729721}\delta^{10}+\frac{17389}{61047}\delta^{8}-\frac{27905594}{43648605}\delta^{6}+\frac{6270226}{8729721}\delta^{4}-\frac{5876077}{14549535}\delta^{2}+\frac{1}{11}\right), \end{array}$$

where *δ*=1+*q*.

A comparison of these results show that all the above solutions are equivalent up to the quadrupole moment. Indeed, a simple redefinition of the parameter *q* which enters all the metrics leads to equivalent values for the monopole and quadrupole moments. We see, however, that differences appear between all the solutions at the level of higher moments. The particularity of the first and second Hernández–Martín solutions is that by choosing the form of the metric *ψ*_*HM*_ appropriately, the multipoles $M_{4}$ and $M_{6}$ can be made to vanish identically. This means that, by following the same procedure, it is possible to generate a solution with only monopole and quadrupole moments. In all the remaining solutions, contributions of higher multipoles are always present.

We conclude that from the point of view of the monopole–quadrupole structure all the solutions presented in §3 are physically equivalent.

## 6. Conclusion

In this work, we analysed all the exact solutions of Einstein’s vacuum field equations which contain the Schwarzschild solution as a particular case and, in addition, possess an arbitrary parameter which determines the quadrupole of the gravitational source. In particular, we studied the Erez–Rosen, Gutsunayev–Manko, Manko, Hernández-Pastora solutions and the *q*-metric, obtained from the Schwarzschild by applying a Zipoy–Voorhees transformation.

First, we established that all the above solutions are asymptotically and elementary flat. This means that at infinity the gravitational field strength is negligible, and the rotation axis is free of conical singularities, respectively. We performed also a detailed analysis of the Kretschmann scalar to determine the curvature singularity structure of these space–times. We found that in general there are three types of naked singularities which are located at the origin of coordinates *r*=0, between the origin and the Schwarzschild horizon r=m(1±cos⁡θ) and on the horizon *r*=2*m*, where *m* is the mass of the gravitational source. The main difference is that only in the case of the *q*-metric, the outermost singularity located at *r*=2*m* exists for all values of the parameters *m* and *q* and the coordinate *θ*. For all the remaining metrics, the second and third singularities exist only for certain specific values of *q* or *θ*. This means that, in principle, it is possible to observe the interior singularities located at *r*=0 and r=m(1±cos⁡θ), which is not possible in the case of a space–time described by the *q*-metric. Suppose that we want to use an interior solution to ‘cover’ the naked singularities generated by the quadrupole. In the case of the *q*-metric, the surface of the interior mass distribution can be located anywhere outside the outermost singularity situated at *r*=2*m*. In the case of all the remaining exterior metrics, the surface of the interior distribution can have even a zero radius for certain values of the quadrupole parameter.

The study of the multipole moments of all the solutions shows that by choosing the quadrupole parameter appropriately all of them are characterized by the same mass and quadrupole, although differences can appear at the level of higher multipoles. This means that all the solutions can be used to describe the exterior gravitational field of a distorted mass distribution with quadrupole moment.

Our results show that all the solutions analysed in this work are equivalent from the physical point of view in the sense that they satisfy all the conditions that are necessary to describe the exterior gravitational field of realistic compact objects. Nevertheless, from a practical point of view the *q*-metric presents certain advantages over the remaining metrics. Indeed, the mathematical structure of this metric is very simple which facilitates its study. For instance, when searching for interior solutions with quadrupole that could be matched with an exterior quadrupolar metric, one certainly would try first the *q*-metric because of its simplicity.

To completely describe the gravitational field of realistic compact objects with quadrupole, it is necessary to take the rotation into account. Moreover, a suitable interior solution is also necessary in order to describe the entire space–time, as required in general relativity. In particular, the specification of an appropriate relativistic matter model is essential in order to end up with a well-posed mathematical problem [30]. Owing to the mathematical complexity of the inner field equations and the matching conditions, it would be easier to start with the simplest possible case which can be handled analytically. Our results show that the *q*-metric is the best candidate for this task. We expect to explore this problem in future works.

## Data accessibility

This article has no additional data.

## Appendix A. The function *γ* for metrics with quadrupole

In the case of the Erez–Rosen metric, the function *γ* takes the form

A 1 $$\begin{array}{rl} \gamma_{\mathrm{ER}} & =\frac{1}{2}{\left(q+1\right)}^{2}\ln\;\left(\frac{x^{2}-1}{x^{2}-y^{2}}\right)-\frac{3}{2}q(1-y^{2})[\frac{3}{32}q(x^{2}-1)(9x^{2}y^{2}-x^{2}-y^{2}+1)\ln^{2}\left(\frac{x-1}{x+1}\right)\\ & \;+\frac{1}{8}x(27qx^{2}y^{2}-3qx^{2}-21qy^{2}+5q+8)\ln\;\left(\frac{x-1}{x+1}\right)+\frac{1}{8}(27qx^{2}y^{2}-3qx^{2}-12qy^{2}+4q+16)]. \end{array}$$

For the Gutsunayev–Manko solution, the function *γ* can be expressed as

A 2 $$\begin{array}{rl} \gamma_{\mathrm{GM}} & =\frac{1}{2}\ln\;\left(\frac{x^{2}-1}{x^{2}-y^{2}}\right)+\frac{1}{2}q\frac{1-y^{2}}{{\left(x^{2}-y^{2}\right)}^{4}}(3(-5y^{2}+1){\left(x^{2}-y^{2}\right)}^{2}+8y^{2}(-5y^{2}+3)(x^{2}-y^{2})\\ & \;+24y^{4}(-y^{2}+1))+\frac{1}{8}q^{2}\frac{\left(1-y^{2}\right)}{{\left(x^{2}-y^{2}\right)}^{8}}(-12(25y^{4}-14y^{2}+1){\left(x^{2}-y^{2}\right)}^{5}\\ & \;+3(-675y^{6}+697y^{4}-153y^{2}+3){\left(x^{2}-y^{2}\right)}^{4}+32y^{2}(-171y^{6}+259y^{4}-105y^{2}+9){\left(x^{2}-y^{2}\right)}^{3}\\ & \;+32y^{4}(-225y^{6}+451y^{4}-271y^{2}+45){\left(x^{2}-y^{2}\right)}^{2}+2304y^{6}(-2y^{6}+5y^{4}-4y^{2}+1)(x^{2}-y^{2})\\ & \;+1152y^{8}(-y^{6}+3y^{4}-3y^{2}+1)). \end{array}$$

The Manko solution [21] must be complemented with the function

A 3 $$\begin{array}{rl} \gamma_{M} & =\frac{1}{2}\ln\;\left(\frac{x^{2}-1}{x^{2}-y^{2}}\right)+\frac{qx(2x^{4}-5x^{2}+5x^{2}y^{2}+3-3y^{2})}{{\left(x^{2}+y^{2}-1\right)}^{5/2}}-2q\\ & \;+\frac{3q^{2}}{8{\left(x^{2}+y^{2}-1\right)}^{5}}\left(\frac{x^{2}y^{2}{\left(5x^{2}y^{2}-3x^{2}-3y^{2}+3\right)}^{2}}{x^{2}+y^{2}-1}-{\left(3x^{2}y^{2}-x^{2}-y^{2}+1\right)}^{2}\right). \end{array}$$

The Hernández-Pastora–Martín solutions are complemented by the following *γ* functions:

A 4 $$\begin{array}{rl} \gamma_{\mathrm{HM}1} & =\frac{1}{2}\left(1+\frac{225}{24}q^{2}\right)\ln\;\left(\frac{x^{2}-1}{x^{2}-y^{2}}\right)-\frac{15}{8}qx(1-y^{2})\\ & \;\times \left(1-\frac{15}{32}q\left[x^{2}+7y^{2}-9x^{2}y^{2}+1-\frac{8}{3}\frac{x^{2}+1}{x^{2}-y^{2}}\right]\right)\ln\;\left(\frac{x-1}{x+1}\right)\\ & \;+\frac{225}{1024}q^{2}(x^{2}-1)(1-y^{2})(x^{2}+y^{2}-9x^{2}y^{2}-1)\ln^{2}\left(\frac{x-1}{x+1}\right)\\ & \;-\frac{15}{4}q(1-y^{2})\left(1-\frac{15}{64}q(x^{2}+4y^{2}-9x^{2}y^{2}+4)\right)-\frac{75}{16}q^{2}x^{2}\frac{1-y^{2}}{x^{2}-y^{2}}-\frac{5}{4}q\frac{\left(x^{2}+y^{2})(1-y^{2}\right)}{{\left(x^{2}-y^{2}\right)}^{2}}\\ & \;-\frac{25}{64}q^{2}(2x^{6}-x^{4}+3x^{4}y^{2}-6x^{2}y^{2}+4x^{2}y^{4}-y^{4}-y^{6})\frac{\left(1-y^{2}\right)}{{\left(x^{2}-y^{2}\right)}^{4}} \end{array}$$

and

A 5 $$\begin{array}{r} \gamma_{\text{HM2}}=\frac{1}{32768}[(\frac{15}{256}q(1-y^{2})\ln\left[\frac{x-1}{x+1}\right](\frac{4x}{{\left(x^{2}-y^{2}\right)}^{3}}(512(8(5A_{1}q-64(x^{2}-y^{2})){\left(x^{2}-y^{2}\right)}^{2}+5A_{2}q^{2})\\ \;-525A_{3}q^{3})-15q(A_{4}q^{2}+A_{5}q+73728x^{2}y^{2}-8192x^{2}-8192y^{2}+8192)(x^{2}-1)\ln\left[\frac{x-1}{x+1}\right])\\ \;+32(223875q^{4}-268800q^{3}+512)\ln\left[\frac{x^{2}-1}{x^{2}-y^{2}}\right])+\frac{5}{64}q(1-y^{2})\\ \;\times (\frac{512}{{\left(x^{2}-y^{2}\right)}^{4}}\left(32(A_{6}q-32(3x^{4}-6x^{2}y^{2}+x^{2}+3y^{4}+y^{2}){\left(x^{2}-y^{2}\right)}^{2})+\frac{5A_{7}q^{2}}{{\left(x^{2}-y^{2}\right)}^{2}}\right)\\ \;-\;\frac{5}{{\left(x^{2}-y^{2}\right)}^{8}}(A_{8}+A_{9}x^{6}y^{2})q^{3})], \end{array}$$

where

$$\begin{array}{rl} A_{1} & =1731x^{4}y^{2}-351x^{4}+1029y^{4}-321y^{2}-16-x^{2}(1731y^{4}+678y^{2}-305)\\ A_{2} & =21x^{6}(5x^{2}(1785x^{2}y^{4}-1020x^{2}y^{2}+51x^{2}-5355y^{6}+850y^{4}+1163y^{2}-82)\\ & \;+26775y^{8}+17850y^{6}-15490y^{4}-450y^{2}+723)\\ & \;-y^{2}(55335y^{8}-24570y^{6}+11505y^{4}-3598y^{2}-240)\\ & \;-3x^{4}(62475y^{10}+196350y^{8}-69160y^{6}-23100y^{4}+14037y^{2}-1146)\\ & \;+x^{2}(232050y^{10}+45675y^{8}-75810y^{6}+38113y^{4}-7036y^{2}+80)\\ A_{3} & =111517875x^{12}y^{6}-116069625x^{12}y^{4}+28676025x^{12}y^{2}-819315x^{12}\\ & \;-334553625x^{10}y^{8}+157794000x^{10}y^{6}+110794950x^{10}y^{4}-45624600x^{10}y^{2}\\ & \;+1461915x^{10}+334553625x^{8}y^{10}+223035750x^{8}y^{8}-413254275x^{8}y^{6}\\ & \;+48671595x^{8}y^{4}+18111870x^{8}y^{2}-691173x^{8}-111517875x^{6}y^{12}\\ & \;-455175000x^{6}y^{10}+288232875x^{6}y^{8}+125092800x^{6}y^{6}-52174485x^{6}y^{4}\\ & \;-567000x^{6}y^{2}+144909x^{6}-91186725x^{2}y^{12}+60618600x^{2}y^{10}+10071750x^{2}y^{8}\\ & \;-6777776x^{2}y^{6}+331047x^{2}y^{4}+107520x^{2}y^{2}+9600x^{2}+10833165y^{12}\\ & \;-10736775y^{10}+2425095y^{8}-146829y^{6}-44160y^{4}+28800y^{2}\\ & \;+x^{4}(190414875y^{12}+76737150y^{10}-198772245y^{8}+33048125y^{6}\\ & \;+5959014y^{4}-498087y^{2}-63360)\\ A_{4} & =11025(354025x^{6}y^{6}-368475x^{6}y^{4}+91035x^{6}y^{2}-2601x^{6}-368475x^{4}y^{6}\\ & \;+379185x^{4}y^{4}-91953x^{4}y^{2}+2907x^{4}+91035x^{2}y^{6}-91953x^{2}y^{4}+22257x^{2}y^{2}\\ & \;-603x^{2}-2601y^{6}+2907y^{4}-603y^{2}+297)\\ A_{5} & =-53760(595x^{4}y^{4}-340x^{4}y^{2}+17x^{4}-340x^{2}y^{4}+212x^{2}y^{2}-16x^{2}+17y^{4}-16y^{2}-1)\\ A_{6} & =13185x^{10}y^{2}-2655x^{10}-52740x^{8}y^{4}+7140x^{8}y^{2}+1440x^{8}+79110x^{6}y^{6}\\ & \;-2010x^{6}y^{4}-5880x^{6}y^{2}+112x^{6}-52740x^{4}y^{8}-10260x^{4}y^{6}+9000x^{4}y^{4}\\ & \;-312x^{4}y^{2}+40x^{4}-3480y^{10}+1560y^{8}+152y^{6}+40y^{4}\\ & \;+x^{2}y^{2}(13185y^{8}+11265y^{6}-6120y^{4}-272y^{2}+240)\\ A_{7} & =562275x^{16}y^{4}-321300x^{16}y^{2}+16065x^{16}-3373650x^{14}y^{6}+1419075x^{14}y^{4}\\ & \;+211050x^{14}y^{2}-20475x^{14}+8434125x^{12}y^{8}-1767150x^{12}y^{6}-1439445x^{12}y^{4}\\ & \;+90930x^{12}y^{2}+78708x^{12}-11245500x^{10}y^{10}-1204875x^{10}y^{8}+3412080x^{10}y^{6}\\ & \;-144165x^{10}y^{4}-451620x^{10}y^{2}+1056x^{10}+57120y^{16}-3360y^{14}+56640y^{12}\\ & \;-1088y^{10}-608y^{8}-160y^{6}-x^{2}y^{4}(508725y^{12}+142380y^{10}-28245y^{8}\\ & \;+361428y^{6}-3648y^{4}+832y^{2}+2400)+3x^{8}(2811375y^{12}+1785000y^{10}\\ & \;-1326675y^{8}+24500y^{6}+358040y^{4}-96y^{2}-96)+x^{4}y^{2}(562275y^{14}+2731050y^{12}\\ & \;-436275y^{10}-70350y^{8}+959940y^{6}-2208y^{4}+10880y^{2}-2400)\\ & \;-x^{6}(3373650y^{14}+5703075y^{12}-2301810y^{10}-45675y^{8}+1356360y^{6}+6240y^{4}-1088y^{2}+160)\\ A_{8} & =35128130625x^{22}y^{6}-36561931875x^{22}y^{4}+9032947875x^{22}y^{2}-258084225x^{22}\\ & \;-281025045000x^{20}y^{8}+244224146250x^{20}y^{6}-22451640750x^{20}y^{4}\\ & \;-10070345250x^{20}y^{2}+374475150x^{20}+983587657500x^{18}y^{10}-637563622500x^{18}y^{8}\\ & \;-129817114875x^{18}y^{6}+73875625425x^{18}y^{4}+848869875x^{18}y^{2}-115835265x^{18}\\ & \;-1967175315000x^{16}y^{12}+695871540000x^{16}y^{10}+762842241000x^{16}y^{8}\\ & \;-198318141000x^{16}y^{6}-19428973200x^{16}y^{4}+718389000x^{16}y^{2}+28304640x^{16}\\ & \;+2458969143750x^{14}y^{14}+143858058750x^{14}y^{12}-1715997701250x^{14}y^{10}\\ & \;+220631836950x^{14}y^{8}+79818927300x^{14}y^{6}-1466001180x^{14}y^{4}-266353920x^{14}y^{2}\\ & \;-29516544x^{14}-1967175315000x^{12}y^{16}-1331523427500x^{12}y^{14}\\ & \;+2098661449500x^{12}y^{12}+36939968100x^{12}y^{10}-165043084500x^{12}y^{8}+222283320x^{12}y^{6}\\ & \;+974776320x^{12}y^{4}+163676160x^{12}y^{2}+410112x^{12}+983587657500x^{10}y^{18}\\ & \;+1679459197500x^{10}y^{16}-1433634441750x^{10}y^{14}-400459377150x^{10}y^{12}\\ & \;+200621102850x^{10}y^{10}+3991429050x^{10}y^{8}-1901034240x^{10}y^{6}-348526848x^{10}y^{4}\\ & \;+4475904x^{10}y^{2}-261120x^{10}-281025045000x^{8}y^{20}-1059101190000x^{8}y^{18}\\ & \;+440141373000x^{8}y^{16}+499964434200x^{8}y^{14}-146690964000x^{8}y^{12}-7673340360x^{8}y^{10}\\ & \;+2224588800x^{8}y^{8}+299837184x^{8}y^{6}-7544064x^{8}y^{4}-1839360x^{8}y^{2}-57600x^{8}\\ & \;-48271308750x^{4}y^{22}-76235118750x^{4}y^{20}+92851469550x^{4}y^{18}-8613319050x^{4}y^{16}\\ & \;-3798768120x^{4}y^{14}+780702720x^{4}y^{12}-257951232x^{4}y^{10}+5118976x^{4}y^{8}\\ & \;+12577280x^{4}y^{6}-4032000x^{4}y^{4}+15755882625x^{2}y^{22}-9452779875x^{2}y^{20}\\ & \;-2139220125x^{2}y^{18}+1125993855x^{2}y^{16}-226571520x^{2}y^{14}+169069824x^{2}y^{12}\\ & \;+2873344x^{2}y^{10}-3266560x^{2}y^{8}-1612800x^{2}y^{6}-815673600y^{22}+719712000y^{20}\\ & \;-147974400y^{18}+31799040y^{16}-37271808y^{14}-284928y^{12}-344320y^{10}-57600y^{8}\\ A_{9} & =35128130625y^{20}+349608538125y^{18}+51702123375y^{16}-304888933125y^{14}\\ & \;+59532473700y^{12}+7143824100y^{10}-1646211840y^{8}+48056064y^{6}-27167744y^{4}\\ & \;+15252480y^{2}-1612800. \end{array}$$

## Appendix B. The Kretschmann scalar of the Erez–Rosen space–time

For the general metric in prolate spheroidal coordinates (2.7), the Kretschmann scalar can be represented as

B 1 $$K=\frac{48}{\sigma^{4}}\frac{P(x,y;\partial \psi ,\partial^{2}\psi ,\partial \gamma ,\partial^{2}\gamma )}{\left(x^{2}-1){\left(x^{2}-y^{2}\right)}^{3}(1-y^{2}\right)}\;e^{4(\psi -\gamma )},$$

where P is a polynomial function in each of its arguments, and ∂^*m*^*ψ* and ∂^*m*^*γ* represent the *m*-th derivatives of *ψ* and *γ* with respect to *x* and *y*. The field equations for *γ* can be used to express all the first and second derivatives of *γ* in terms of those of *ψ*. Using these relations, the Kretschmann scalar becomes

B 2 $$K=\frac{48}{\sigma^{4}}\frac{P(x,y;\partial \psi ,\partial^{2}\psi )}{{\left(x^{2}-y^{2}\right)}^{4}}\;e^{4(\psi -\gamma )},$$

where *P* is also a polynomial function in each of its arguments. The explicit forms of the polynomial functions P and *P* are not given here because they involve very long expressions which do not provide any insight for the present analysis. We see that, when written in this form, the Kretschmann scalar shows only one singularity when *x*^2^−*y*^2^=0. Nevertheless, the behaviour of the polynomial function and the exponential factor could cancel this divergence or introduce new ones.

For metrics whose function *ψ* depends on both *x* and *y*, the analysis of the Kretschmann scalar reduces to the analysis of the polynomial *P*(*x*,*y*,∂*ψ*,∂^2^*ψ*) and the exponential *e*^4(*ψ*−*γ*)^ and their relationship with the factor (*x*^2^−*y*^2^)^−4^. The Schwarzschild metric serves as a guide mark to see what kind of behaviour to expect from the polynomial and exponential factors in *K*. In this case, the metric functions are given by equations (2.12) that correspond to *e*^4(*ψ*−*γ*)^=(*x*+1)^−4^(*x*^2^−*y*^2^)^2^ and *P*(*x*,*y*;∂*ψ*,∂^2^*ψ*)=(*x*+1)^−2^(*x*^2^−*y*^2^)^2^, leading to the scalar (4.3). This means that both the exponential and the polynomial factors contribute to generate the divergence at *x*=−1, and also to cancel out the original factor that diverges at *x*=±*y*.

In the case of other static axisymmetric metrics, the exponential and the polynomial factors produce new divergent factors or factors that cancel the original divergent factor. Let us consider in detail the case of the Erez–Rosen metric. The exponential and polynomial factors are

B 3 $$e^{4(\psi -\gamma )}=\frac{\exp[\sum_{n=0}^{2}\pi_{n}(x,y;q){\left(\ln(x-1)/(x+1)\right)}^{n}]}{{\left(x+1\right)}^{2(q^{2}+2q+2)}{\left(x-1\right)}^{2q(q+2)}{\left(x^{2}-y^{2}\right)}^{-2{\left(q+1\right)}^{2}}}$$

and

B 4 $$P(x,y;\partial \psi ,\partial^{2}\psi )=\frac{x^{2}-y^{2}}{{\left(x^{2}-1\right)}^{2}}\sum_{n=0}^{6}p_{n}(x,y;q){\left(\ln\frac{x-1}{x+1}\right)}^{n},$$

where *π*_*n*_ and *p*_*n*_ are polynomial functions of each of its arguments with the following properties:

$$\begin{array}{rl} \pi_{n}(x,y;0) & =0,\;\forall n,\\ p_{n}(x,y;q) & =q^{n}{\tilde{p}}_{n}(x,y;q),\;\forall n,\\ \pi_{2}(\pm 1,y;q) & =0,\\ p_{n}(\pm 1,y;q) & =0,\;n>0\\ \;\mathrm{and}\;p_{n}(x=\pm y,y;q) & =0,\;n\geq 4, \end{array}$$

so that the Kretschmann can be written as

B 5 $$K_{\text{ER}}=\frac{48}{\sigma^{4}}\frac{\left[\sum_{n=0}^{6}p_{n}(x,y;q){\left(\ln(x-1)/(x+1)\right)}^{n}]\exp[\sum_{n=0}^{2}\pi_{n}(x,y;q){\left(\ln(x-1)/(x+1)\right)}^{n}\right]}{{\left(x+1\right)}^{2(q^{2}+2q+3)}{\left(x-1\right)}^{2{\left(q+1\right)}^{2}}{\left(x^{2}-y^{2}\right)}^{-2q^{2}-4q+1}}.$$

For the particular cases *q*=0, *q*=−1 and *q*=−2, the factor $\sum_{n=0}^{6}p_{n}(x,y;q)$ ${\left(\ln(x-1)/(x+1)\right)}^{n}$ vanishes for *x*=±1, or *x*=±*y*. Indeed, for *q*=0, we have that $\sum_{n=0}^{6}p_{n}(x,y;0)$ ${\left(\ln((x-1)/(x+1))\right)}^{n}$ =*p*_0_(*x*,*y*;0)=(*x*−1)^2^(*x*^2^−*y*^2^), and using also that *π*_*n*_(*x*,*y*;0)=0, we obtain *K*_ER_|_*q*=0_=(48/*σ*^4^)(1/(*x*+1)^6^), which corresponds to the Kretschmann scalar of the Schwarzschild metric, as expected. For *q*=−1, it turns out that all the polynomials *p*_*n*_(*x*,*y*;−1) have roots at *x*=±*y* with multiplicity two for 0≤*n*≤3 and multiplicity three for *n*≥4. Therefore, the term (*x*^2^−*y*^2^)^2^ is cancelled with the one in the denominator and the Kretschmann scalar turns out to be

B 6 $$K_{\mathrm{ER}}{|}_{q=-1}=\frac{48}{\sigma^{4}}\frac{\left[\sum_{n=0}^{6}{\bar{p}}_{n}(x,y){\left(\ln((x-1)/(x+1))\right)}^{n}]\exp[\sum_{n=0}^{2}\pi_{n}(x,y;-1){\left(\ln((x-1)/(x+1))\right)}^{n}\right]}{{\left(x+1\right)}^{4}(x^{2}-y^{2})}.$$

For *q*=−2, the polynomials *p*_*n*_(*x*,*y*;−2) have roots at *x*=±*y*, but now with multiplicity one for 0≤*n*≤4, multiplicity two for *n*=5 and multiplicity three for *n*=6. Then, in a similar way, the term *x*^2^−*y*^2^ is cancelled so that

B 7 $$K_{\text{ER}}{|}_{q=-2}=\frac{48}{\sigma^{4}}\frac{\left[\sum_{n=0}^{6}{\bar{\bar{p}}}_{n}(x,y){\left(\ln((x-1)/(x+1))\right)}^{n}]\exp[\sum_{n=0}^{2}\pi_{n}(x,y;-2){\left(\ln((x-1)/(x+1))\right)}^{n}\right]}{{\left(x+1\right)}^{6}{\left(x-1\right)}^{2}}.$$

For values of *q*, different from the ones analysed above, the Kretschmann scalar for the Erez–Rosen metric presents divergences at *x*=±1 and *x*=±*y*, if the exponents of the corresponding factors are negative and if the exponential factor does not vanish for those values, in which case one would have to calculate the limit explicitly. The exponent of the factors *x*−1 has a negative definite sign and so there is a curvature singularity at *x*=−1. On the contrary, the exponent of the factor *x*^2^−*y*^2^ vanishes for $q=-1\pm \sqrt{\frac{3}{2}}$, indicating that for certain ranges of values of *q* there could be curvature singularities at *x*=±*y*.

The case *x*=1 deserves a detailed analysis for which we have that

B 8 $${\sum_{n=0}^{6}p_{n}(x,y;q){\left(\ln\frac{x-1}{x+1}\right)}^{n}|}_{x=1}=p_{0}(1,y;q)$$

and

B 9 $${\sum_{n=0}^{2}\pi_{n}(x,y;q){\left(\ln\frac{x-1}{x+1}\right)}^{n}|}_{x=1}=\pi_{0}(1,y;q)+{\pi_{1}(x,y;q)\ln\frac{x-1}{x+1}|}_{x=1},$$

so that taking the limit, we obtain

B 10 $$\lim_{x\to 1}K_{\text{ER}}=\frac{48}{\sigma^{4}}\frac{p_{0}(1,y;q)\;e^{\pi_{0}(1,y;q)}}{2^{2(q^{2}+2q+3)+\pi_{1}(1,y;q)}{\left(1-y^{2}\right)}^{-2q^{2}-4q+1}}\lim_{x\to 1}{\left(x-1\right)}^{\pi_{1}(1,y;q)-2{\left(q+1\right)}^{2}}.$$

This limit vanishes for *π*_1_(1,*y*;*q*)−2(*q*+1)^2^≥0 and diverges for *π*_1_(1,*y*;*q*)−2(*q*+1)^2^<0. Explicitly *π*_1_ is given by $\pi_{1}(1,y;q)=\frac{3}{2}q\{q[1+3y^{2}(\frac{2}{3}-y^{2})]+2(\frac{5}{3}-y^{2})\}$ so that the exponent can be written as $\pi_{1}(1,y;q)-2{\left(q+1\right)}^{2}=-\frac{1}{2}({\left[q(3y^{2}-1)+1\right]}^{2}+3),$ which is strictly negative, meaning the Kretschmann scalar diverges for *x*=1. The sign of the divergence will be determined by *p*_0_(1,*y*;*q*), which explicitly is given by $p_{0}(1,y;q)=\frac{1}{192}q^{2}(1-y^{2}){\left(3y^{2}-1\right)}^{2}{\left(q[3y^{2}-1]+2\right)}^{2}({\left[q(3y^{2}-1)+1\right]}^{2}+3)$, which given the range of *y*, *y*∈(−1,1), is positive definite, hence

B 11 $$\lim_{x\to 1}K_{\mathrm{ER}}=+\infty .$$

We thus conclude that the Erez–Rosen space–time has a singularity at *x*=−1, independently of the value of *q* and the coordinate *y*. Then, for certain values of *q*, there is a second singularity at *x*=±*y* and, finally, at *x*=1 there could be a third singularity, depending on the value of *q*. All these singularities are naked in the sense that they are not covered by an exterior horizon. This means that, for particular values of *q*, it is possible to observe the singularity located at the origin of coordinates *x*=−1.

## Appendix C. Explicit expressions for the multipole moments

The multipole moments *M*_*n*_ for a given solution can be obtained from the derivatives of the Ernst potential evaluated at the axis of symmetry, *m*_*n*_, plus an additional term *d*_*n*_ which is different for each *n* and can be expressed in terms of *m*_*n*_. The additional terms for *n*=1,…,10 are expressed as

C 1 $$\begin{array}{rl} d_{0} & =d_{1}=d_{2}=d_{3}=0,\\ d_{4} & =\frac{1}{7}m_{0}(m_{1}^{2}-m_{2}m_{0}),\\ d_{5} & =\frac{1}{3}m_{0}(m_{2}m_{1}-m_{3}m_{0})+\frac{1}{21}m_{1}(m_{1}^{2}-m_{2}m_{0}),\\ d_{6} & =\frac{2}{11}m_{0}(m_{3}m_{1}-3m_{4}m_{0})+\frac{1}{33}m_{0}^{3}(m_{2}m_{0}+m_{1}^{2})+\frac{1}{77}m_{2}(11m_{1}^{2}+17m_{0}m_{2}),\\ d_{7} & =\frac{1}{39}m_{0}(18m_{3}m_{2}-33m_{5}m_{0}-4m_{2}m_{1}m_{0}^{2})\\ & \;+\frac{1}{429}(12m_{4}m_{1}m_{0}+51m_{3}m_{1}^{2}+45m_{3}m_{0}^{4}+69m_{2}^{2}m_{1}-m_{1}^{3}m_{0}^{2}),\\ d_{8} & =-m_{6}m_{0}^{2}+\frac{1}{39}(3m_{4}m_{1}^{2}+m_{0}(9m_{4}m_{0}^{3}-6m_{5}m_{1}+11m_{3}^{2}))\\ & \;+\frac{1}{429}(m_{0}(180m_{4}m_{2}-36m_{3}m_{1}m_{0}^{2}-3m_{2}m_{0}^{5}+3m_{1}^{2}m_{0}^{4})+m_{2}(138m_{3}m_{1}+23m_{2}^{2}))\\ & \;+\frac{1}{3003}m_{0}(31m_{1}^{4}-382m_{2}^{2}m_{0}^{2}-90m_{2}m_{1}^{2}m_{0}),\\ d_{9} & =\frac{1}{17}m_{0}(7m_{5}m_{0}^{3}-21m_{7}m_{0}-6m_{6}m_{1})+\frac{1}{11}m_{2}\left(\frac{23}{13}m_{3}m_{2}+\frac{6}{17}m_{1}m_{0}^{5}\right)\\ & \;+\frac{1}{221}(76m_{5}m_{2}m_{0}+5m_{5}m_{1}^{2}+64m_{4}m_{2}m_{1}+4m_{4}m_{1}m_{0}^{3}-80m_{3}m_{2}m_{0}^{3}-7m_{3}m_{0}^{6})\\ & \;+\frac{1}{2431}(1432m_{4}m_{3}m_{0}+443m_{3}^{2}m_{1}-126m_{3}m_{1}^{2}m_{0}^{2}-m_{1}^{3}m_{0}^{4})\\ & \;+\frac{1}{17017}m_{1}(41m_{1}^{4}-1002m_{2}^{2}m_{0}^{2}+688m_{2}m_{1}^{2}m_{0})\\ \;\text{and}\;d_{10} & =\frac{1}{323}(210m_{6}m_{0}^{4}-476m_{8}m_{0}^{2}-182m_{7}m_{1}m_{0}+80m_{6}m_{2}m_{0}-13m_{6}m_{1}^{2}\\ & \;+70m_{5}m_{1}m_{0}^{3}-28m_{4}m_{0}^{6})+\frac{1}{4199}(2406m_{5}m_{3}m_{0}+982m_{5}m_{2}m_{1}+699m_{3}^{2}m_{2}\\ & \;+126m_{3}m_{1}m_{0}^{5}+7m_{2}m_{0}^{8}-7m_{1}^{2}m_{0}^{7})+\frac{1}{13}m_{1}^{4}\left(\frac{205}{1309}m_{2}-\frac{50}{969}m_{0}^{3}\right)\\ & \;+\frac{1}{46189}(15319m_{4}^{2}m_{0}+17198m_{4}m_{3}m_{1}+7039m_{4}m_{2}^{2}-19406m_{4}m_{2}m_{0}^{3}\\ & \;-1439m_{4}m_{1}^{2}m_{0}^{2}-10942m_{3}m_{2}m_{1}m_{0}^{2})+\frac{1}{138567}m_{0}(3700m_{3}m_{1}^{3}-39317m_{3}^{2}m_{0}^{2}\\ & \;+7589m_{2}^{2}m_{0}^{4}+815m_{2}m_{1}^{2}m_{0}^{3})+\frac{1}{969969}m_{2}^{2}m_{0}(66930m_{1}^{2}-2609m_{2}m_{0}). \end{array}\}$$
